# The INCREASE project: Intelligent Collections of food‐legume genetic resources for European agrofood systems

**DOI:** 10.1111/tpj.15472

**Published:** 2021-09-23

**Authors:** Elisa Bellucci, Orlando Mario Aguilar, Saleh Alseekh, Kirstin Bett, Creola Brezeanu, Douglas Cook, Lucía De la Rosa, Massimo Delledonne, Denise F. Dostatny, Juan J. Ferreira, Valérie Geffroy, Sofia Ghitarrini, Magdalena Kroc, Shiv Kumar Agrawal, Giuseppina Logozzo, Mario Marino, Tristan Mary‐Huard, Phil McClean, Vladimir Meglič, Tamara Messer, Frédéric Muel, Laura Nanni, Kerstin Neumann, Filippo Servalli, Silvia Străjeru, Rajeev K. Varshney, Marta W. Vasconcelos, Massimo Zaccardelli, Aleksei Zavarzin, Elena Bitocchi, Emanuele Frontoni, Alisdair R. Fernie, Tania Gioia, Andreas Graner, Luis Guasch, Lena Prochnow, Markus Oppermann, Karolina Susek, Maud Tenaillon, Roberto Papa

**Affiliations:** ^1^ Department of Agricultural, Food and Environmental Sciences Polytechnic University of Marche via Brecce Bianche Ancona 60131 Italy; ^2^ Instituto de Biotecnología y Biología Molecular UNLP‐CONICET CCT La Plata La Plata Argentina; ^3^ Max‐Planck‐Institute of Molecular Plant Physiology Am Müe Potsdam‐Golm 14476 Germany; ^4^ Centre of Plant Systems Biology and Biotechnology Plovdiv 4000 Bulgaria; ^5^ Department of Plant Sciences University of Saskatchewan 51 Campus Drive Saskatoon SK S7N 5A8 Canada; ^6^ Staţiunea de Cercetare Dezvoltare Pentru Legumicultură Bacău 600388 Romania; ^7^ Department of Plant Pathology University of California Davis Davis CA 95616‐8680 USA; ^8^ Spanish Plant Genetic Resources National Center (INIA, CRF) National Institute for Agricultural and Food Research and Technology Alcalá de Henares Madrid 28800 Spain; ^9^ Department of Biotechnology University of Verona Strada Le Grazie 15 Verona 37134 Italy; ^10^ National Centre for Plant Genetic Resources, Plant Breeding and Acclimatization Institute‐NRI Radzików Błonie 05‐870 Poland; ^11^ Regional Service for Agrofood Research and Development (SERIDA) Ctra AS‐267, PK 19 Villaviciosa Asturias 33300 Spain; ^12^ CNRS INRAE Institute of Plant Sciences Paris‐Saclay (IPS2) Univ Evry Université Paris‐Saclay Orsay 91405 France; ^13^ CNRS INRAE Institute of Plant Sciences Paris Saclay (IPS2) Université de Paris Orsay 91405 France; ^14^ ISEA Srl Via dell’Industria n.303 Corridonia MC 62014 Italy; ^15^ Legume Genomics Team Institute of Plant Genetics Polish Academy of Sciences Strzeszynska 34 Poznan 60‐479 Poland; ^16^ Genetic Resources Section International Center for Agricultural Research in the Dry Areas ICARDA Agdal Rabat Morocco; ^17^ School of Agricultural, Forestry, Food and Environmental Sciences University of Basilicata Potenza 85100 Italy; ^18^ International Treaty on Plant Genetic Resources for Food and Agriculture (ITPGRFA) Food and Agriculture Organization of the United Nations (FAO) Viale delle Terme di Caracalla Rome 00153 Italy; ^19^ INRAE CNRS AgroParisTech Génétique Quantitative et Evolution ‐ Le Moulon Université Paris‐Saclay Gif‐sur‐Yvette France; ^20^ Department of Plant Sciences, Genomics and Bioinformatics Program North Dakota State University Fargo ND 58108 USA; ^21^ Crop Science Department Agricultural Institute of Slovenia Hacquetova ulica 17 Ljubljana 1000 Slovenia; ^22^ EURICE ‐ European Research and Project Office GmbH Heinrich‐Hertz‐Allee 1 St. Ingbert 66386 Germany; ^23^ Terres Inovia Institut Technique des oléagineux, des protéagineux eu du chanvren 1 Av L. Brétignières Thiverval-Grignon 78850 France; ^24^ Leibniz Institute of Plant Genetics and Crop Plant Research (IPK) Gatersleben Seeland 06466 Germany; ^25^ Comunità del Mais Spinato di Gandino (MASP) Via XX Settembre, 5 Gandino Bergamo 24024 Italy; ^26^ Suceava Genebank (BRGV) Bdul 1 Mai, nr. 17 Suceava 720224 Romania; ^27^ Center of Excellence in Genomics and Systems Biology (CEGSB) International Crops Research Institute for the Semi- Arid Tropics (ICRISAT) Patancheru India; ^28^ State Agricultural Biotechnology Centre Centre for Crop and Food Innovation Food Futures Institute Murdoch University Murdoch Western Australia Australia; ^29^ CBQF – Centro de Biotecnologia e Química Fina – Laboratório Associado Escola Superior de Biotecnologia Universidade Católica Portuguesa Rua Diogo Botelho 1327 Porto 4169-005 Portugal; ^30^ Council for Agricultural Research and Economics Research Centre for Vegetable and Ornamental Crops Via Cavalleggeri 25 Pontecagnano‐Faiano SA 84098 Italy; ^31^ Federal Research Center The N.I. Vavilov All‐Russian Institute of Plant Genetic Resources St. Petersburg 190031 Russia; ^32^ Department of Information Engineering Polytechnic University of Marche via Brecce Bianche Ancona 60131 Italy

**Keywords:** plant genetic resources, symbiosis, high‐throughput phenotyping, artificial intelligence, metabolomics

## Abstract

Food legumes are crucial for all agriculture‐related societal challenges, including climate change mitigation, agrobiodiversity conservation, sustainable agriculture, food security and human health. The transition to plant‐based diets, largely based on food legumes, could present major opportunities for adaptation and mitigation, generating significant co‐benefits for human health. The characterization, maintenance and exploitation of food‐legume genetic resources, to date largely unexploited, form the core development of both sustainable agriculture and a healthy food system. INCREASE will implement, on chickpea (*Cicer arietinum*), common bean (*Phaseolus vulgaris*), lentil (*Lens culinaris*) and lupin (*Lupinus albus* and *L. mutabilis*), a new approach to conserve, manage and characterize genetic resources. *Intelligent Collections*, consisting of nested core collections composed of single‐seed descent‐purified accessions (i.e., inbred lines), will be developed, exploiting germplasm available both from genebanks and on‐farm and subjected to different levels of genotypic and phenotypic characterization. Phenotyping and gene discovery activities will meet, via a participatory approach, the needs of various actors, including breeders, scientists, farmers and agri‐food and non‐food industries, exploiting also the power of massive metabolomics and transcriptomics and of artificial intelligence and smart tools. Moreover, INCREASE will test, with a citizen science experiment, an innovative system of conservation and use of genetic resources based on a decentralized approach for data management and dynamic conservation. By promoting the use of food legumes, improving their quality, adaptation and yield and boosting the competitiveness of the agriculture and food sector, the INCREASE strategy will have a major impact on economy and society and represents a case study of integrative and participatory approaches towards conservation and exploitation of crop genetic resources.

## Significance Statement

INCREASE, focusing on important food legumes, i.e., chickpea (*Cicer arietinum*), common bean (*Phaseolus vulgaris*), lentil (*Lens culinaris*) and lupin (*Lupinus albus* and *L. mutabilis*), develops new approaches to conserve, manage and characterize genetic resources. INCREASE is working with Intelligent Collections, consisting of nested core collections composed of single‐seed descent‐purified accessions, exploiting germplasm both from genebanks and on‐farm; these will be subjected to different levels of genotyping and phenotyping in order to meet, via a participatory approach, the needs of various actors, including breeders, scientists, farmers, citizen scientists and agri‐food and non‐food industries, including traits related to the value chain, to agroecosystem characteristics and to adaptation to climate change.

## INTRODUCTION

The characterization and maintenance of food‐legume genetic resources and their exploitation in pre‐breeding form the core development of both more sustainable agriculture and healthier food products. Indeed, in 2019 the IPCC report titled ‘Climate Change and Land’ (https://www.ipcc.ch/report/srccl/) indicated that the transition to novel plant‐based diets could ‘present major opportunities for adaptation and mitigation while generating significant co‐benefits in terms of human health’. Such transition is particularly relevant in the light of recent health concerns regarding the prominence of zoonosis (Broglia and Kapel, 2011). In addition, most legume species can establish symbiotic associations with nitrogen‐fixing bacteria (rhizobia), making them of high economic and ecologic importance (Domínguez et al., 2016; Kakraliya et al., 2018). While reducing the use of chemical fertilizers, they not only benefit from high protein content in their seeds, but they also return reduced nitrogen to the soil, thereby enhancing fertility of agroecosystems’ productivity and sustainability (Gordon et al., 2001; Reckling et al., 2016).

Historically, legumes were a primary source of agricultural nitrogen, because they were grown in rotation with cereals (Preissel et al., 2015). Most of the modern intensive agricultural systems rely instead on nitrogen fertilizers, produced by the Haber–Bosch industrial process, which demands high quantities of non‐renewable fossil fuels to reduce N_2_ to NH_4_ (EU In‐depth Report, 2013, https://ec.europa.eu/environment/integration/research/newsalert/pdf/IR6_en.pdf). Production of industrial fertilizers contributes approximately 3% of global CO_2_ and is a primary source of the pollutant NO_2_ (Wood and Cowie, 2004). Furthermore, runoff from fertilizer is among the world’s most serious environmental pollutants, causing also eutrophication of aquatic ecosystems (Rockström et al., 2009). Therefore, exploiting legume genetic resources to improve the symbiosis between crop legumes and their associated rhizobia could have a major impact on sustainable agriculture and on the world’s economic, social and environmental health.

Almost half of the current global food production depends on planetary boundary transgressions infringing on biosphere integrity, land‐system change, freshwater use and nitrogen flows. As highlighted by Gerten et al. (2020), the transition towards a plant‐based diet is probably the most important opportunity to promote food security and to respect the planetary boundaries. Transition towards plant‐based diets is already underway and human plant protein intake is on the rise in many EU regions; the market for meat and dairy alternatives is particularly promising, with annual growth rates of 14 and 11%, respectively (EC Report COM (2018) 757 final, https://eur‐lex.europa.eu/legal‐content/EN/TXT/?uri=CELEX%3A52018DC0757). This implies that a crucial aspect leading to added value to European and worldwide primary production will be the improvement of nutritional and quality traits, considering the development of innovative products, possibly linked to sustainable agroecosystems, and the local production in line with agricultural and food tradition, which are important factors in consumer preferences. Thus, the challenge is to meet citizens’ needs and preferences (e.g., changing dietary habits) regarding impact on health, environment and climate change mitigation. For this purpose, healthy and environmentally friendly food based on the local production of novel varieties is needed and existing genetic resources must be properly exploited in breeding within sustainable agroecosystems. In addition, the value chain needs re‐enforcing with new varieties with higher adaptation to the environment of cultivation, better yield, and improved qualities such as high organoleptic, technological and nutritional values.

However, especially in the case of food legumes, investment in breeding research has been modest, leading to a largely unexplored genetic potential of these important staple food crops. The European Union devotes only 3% of its arable land to protein crops, and imports more than 75% of its plant protein (e.g., importing each year about 400 000 tonnes of common bean, 200 000 tonnes of lentil and 150 000 tonnes of chickpea; Kezeya Sepngang et al., 2020; EC Report A8‐0121/2018). The low level of European plant protein self‐sufficiency is due to the late development and poor adaptation of protein plants in Europe (COPA‐COGECA report, GOL (18)585), as well to the lack of breeding efforts for adaptation of legumes to European agro‐ecosystems. The exploitation of genetic resources in food legume breeding is limited in comparison to the availability of materials, and consequently the potential impact of their use is far from optimal (i.e., lack of comprehensive information regarding descriptive metadata, in particular so‐called passport data, and descriptors valuable for users, accession heterogeneity, unharmonized data), which also affects the ability to attract funds for genetic‐resource conservation. These issues are particularly acute in food legumes, as breeding investment and research activities remain modest, thus their genetic potential results to be unexplored. While these aspects limit the actual use of food‐legume genetic resources, the same observation indicates that the marginal return of investment in legume research is likely to be much higher than in other species, where research has been much more intensive and genetic potential has been extensively explored (as in cereal crops), but where crop improvement is now stagnating (Ray et al., 2012; Semba et al., 2021).

National activities and recent projects are now growing due to stimulus of consumer demand and market trends not only in the EU but also worldwide (Clément et al., 2018; Pilorge and Muel, 2016; Watson et al., 2017). Alternative plant proteins for food are demanded (*EU Agricultural Outlook for markets and income 2018–2030*, https://ec.europa.eu/info/sites/default/files/food‐farming‐fisheries/farming/documents/medium‐term‐outlook‐2018‐report_en.pdf) and the EU has developed a new protein plan (Clément et al., 2018), whose implementation will be largely based on traditional and innovative uses of food legumes reflecting the high interest of the food industry and agricultural sector in development of products to meet consumer requests for healthy diets.

**Figure 1:**
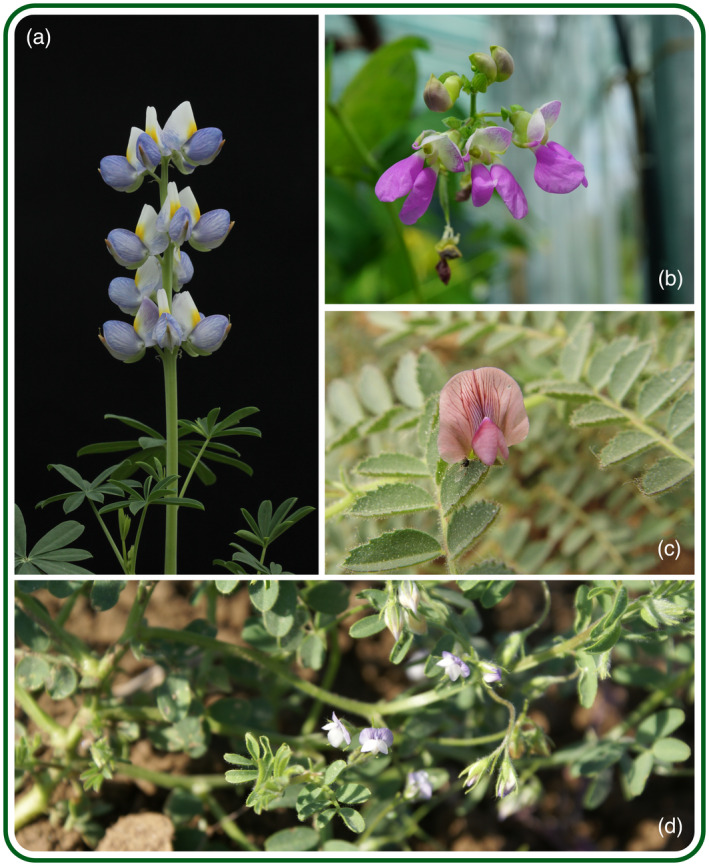
Flowers of the four INCREASE legume species. (a) Lupin (*Lupinus mutabilis* Sweet). (b) Common bean (*Phaseolus vulgaris* L.). (c) Chickpea (*Cicer arietinum* L.). (d) Lentil (*Lens culinaris* Medik.).

To respond to this high‐priority demand, the INCREASE project will implement a new approach to conserve, manage and characterize genetic resources of four major food legumes: chickpea, common bean, lentil and lupin (Figure 1), leading to multi‐level benefits and promising to attract additional private and public investment to boost food legume breeding.

The purpose of INCREASE is to facilitate access to well‐described and well‐managed collections of genetic resources capturing the full range of species, which is of crucial importance for reaching a competitive level regarding agronomic performance and sustainability in the EU. The availability of novel varieties will also facilitate the adoption of food legumes within the agroecosystems, improving the agrobiodiversity with all its related positive consequences associated with the inclusion of legumes in the cropping system (sustainability, food security, economic returns, stable farming systems, soil fertility, diversification of products, etc.), being also in line with the Sustainable Development Goal 15.

Thus, efficient and cutting‐edge genetic resource management is required to attract additional private and public investment. Without the correct handling of legume genetic resources, indeed, the development of sustainable agriculture to match economic, environmental, climatic and socio‐economic challenges including the promotion of healthier diets will be unattainable.

## A STRATEGY FOR PLANT GENETIC RESOURCES FOR FOOD AND AGRICULTURE CONSERVATION AND UTILIZATION

The limited actual utilization of plant genetic resources in comparison to the availability of materials and the potential impact of their use is due to several concurrent factors:

(i) the genetic structure of accessions; in most cases, accessions have an unknown genetic structure and are heterogeneous, which impedes the projection of the phenotypic information to the genotype and *vice versa*; moreover, if available, single‐nucleotide polymorphism data are mapped to a single reference genome, which exclude variants present only in a subset of accessions, without the possibility to access structural variations and to obtain a complete picture of the functional genetic variation (Richard et al., 2021);
(ii) limited information on plant genetic resources and data quality; passport data (e.g., biological status and geographic origin) are often incomplete and phenotypic evaluations are often limited to morphological and a few major agronomic traits;
(iii) limited access to available information; the heterogeneous nature and non‐standardized (biased) way of data collection and integration cause an under‐use of the huge amount of existing information and data; databases are centralized, not interconnected and not designed to integrate data obtained by external users, strongly limiting the access to available information, which remains separated in genebank/project information systems; also, the available information is not easily accessible to users due to unfriendly searching and visualization tools (Krajewski et al., 2015).

The optimal use of genetic resources represents a precondition to increase sustainability. However, their *ex situ* conservation is based on empirical principles which were defined over 50 years ago, and *on‐farm* conservation activities are only marginally developed, the latter being often disconnected from genebank initiatives (Díez et al., 2018; ECPGR, 2017; Maxted et al., 2002; Rao, 2009; Thomas et al., 2011, 2012a, 2012b). Seed collections are assembled and maintained on an accession‐by‐accession basis, whereby each accession often comprises a mixture (population) with an unknown composition of genotypes. Conservation of these heterogeneous accessions raises substantial challenges due to the genetic changes that can occur during seed multiplication in genebanks (i.e., genetic drift and/or selection). Handling these processes requires to monitor neutral and functional diversity at both the genetic and phenotypic levels, which means: characterizing it, investigating its interactions with the environment to define useful functional variation in given environments and establishing a link between variation of phenotypes and ‘biomarkers’ to predict the potential utility of accessions for specific breeding goals.

Based on technological advances, the abovementioned shortcomings regarding both conservation management and activation of collections for research and plant breeding can be systematically addressed. Recently, major advances have been made in the development of single‐seed descent (SSD)‐purified accessions, bringing with it the possibility to better associate phenotypes to reliable genotypic information (Milner et al., 2019; Russell et al., 2016; Sansaloni et al., 2020; Varshney et al., 2019). This shows a strong potential to promote genetic resources and their use in pre‐breeding programs (De la Rosa et al., 2021; Riaz et al., 2017).

The choice of legume species in the INCREASE project represents a cross‐section in terms of their potential value for sustainable food production, and they are all strongly linked to the European food tradition and needs, thereby being of considerable importance for EU agriculture. These species represent extremes regarding their genome sizes and provide a comprehensive panoply of genomic resources. For these crops, INCREASE is implementing a new approach to conserve, manage and characterize genetic resources, with integration of the data produced. We will make the information freely available in order to develop golden standards for data sharing and exploitation. As such, we hope that INCREASE will represent a step towards a coordinated, interdisciplinary and multi‐sectorial effort which is needed to exploit the recent scientific and technological groundbreaking advances, ranging from genomics to information technology (IT) and artificial intelligence (AI), in order to develop an innovative platform for conservation and sustainable use of genetic resources.

## INCREASE GROUNDS

The general objective of INCREASE is to improve the sustainable use of plant genetic resources in food and agriculture working with the four abovementioned important food legumes and promoting the conservation and use of all kinds of their genetic resources. The INCREASE goal will be achieved through the improvement of food‐legume genetic resource data management and sharing, by development of optimized databases, data‐management solutions and web‐based searching and data visualization tools, based on data findability, accessibility, interoperability and reusability (FAIR) principles to facilitate, with much more useful and easily accessible information, the access of stakeholders to plant genetic resources (Ghaffar et al., 2019; Halewood et al., 2018; König et al., 2020; Neveu et al., 2019; Van Treuren and Van Hintum, 2014). The joint and integrated work of the INCREASE consortium will produce massive and high‐quality genotypic and phenotypic data using cutting‐edge methodologies (McCouch et al., 2020) obtained during the project or/and already established by INCREASE partners. The development of new knowledge (e.g., gene discovery, genomic prediction), easily available for stakeholders by user‐friendly web‐based searching and visualization tools (Crossa et al., 2016), and, in parallel, the development, testing and dissemination of best practices for dynamic management of plant genetic resources across European and non‐European institutions and initiatives will promote and enhance the sustainable use of plant genetic resources, providing an efficient possibility to identify the appropriate source of germplasm with minor effort and to meet manifold requirements of users by targeting traits of interest for adaptation to European agro‐ecosystems and for the agri‐food and non‐food industries and by participatory approaches.

INCREASE will additionally enrich the information management of genebanks by facilitating the communication and coordination between different institutions and by promoting data collection and process management and data sharing solutions, in both a centralized and decentralized manner; the access to users will be facilitated through a participatory strategy and this will allow the design of innovative conservation plans, which can include decentralized management approaches. To reach these goals, INCREASE is planning to enhance the efficiency, standardization and speed of data handling and facilitate the integration of information produced by users that, presently, cannot be integrated in databases due to problems of data compatibility and cost accrued on data input, which consequently limit the availability of relevant data for (and from) stakeholders. Moreover, citizen science activities will connect farmers and consumers with the plant genetic resource conservation system to raise awareness in the general public and to stimulate the use of plant genetic resources among different stakeholders and to test a decentralized solution on data and germplasm sharing. INCREASE will, furthermore, acquire comprehensive and more precise genotypic and phenotypic information on materials, to improve our understanding of the connections between them and how they vary in different environmental contexts; the project will put in place appropriate (bioinformatics) tools for data processing, exchange and visualization. INCREASE will characterize the plant genetic resources of the four different food legumes, and a representative sample of diversity will be developed for each one, including wild and domesticated accessions, that will be characterized at the genotypic and phenotypic levels using a combination of ‘‐omics’ tools, image analysis and field phenotyping.

Using an interdisciplinary approach, INCREASE aims to implement and improve data management solutions and visualization tools to facilitate user access, also involving different users and stakeholders in the dynamic management and conservation. Thus, INCREASE will pave the way for informed use of plant genetic resources based on the user and stakeholder inventory needs.

### INCREASE Intelligent Collections

In order to efficiently explore the plant genetic resources diversity, for each crop, INCREASE will assemble and curate *Intelligent Collections* (ICs, *Intelligent* as in able to *memorize*, *learn*, *improve* and *evolve*) as a set of nested core collections of different sizes that represent the entire diversity of each crop (Cortinovis et al., 2021).

On the basis of what was developed during the project BEAN_ADAPT (ERA‐CAPS 2nd call, 2014), for each crop, ICs will be developed, a set of nested core collections of genetically purified accessions (based on single homozygous genotypes, purified by one or two cycles of SSD) from a large sample of representative accessions from genebanks and from dynamic conservation on‐farm. ICs will: (i) have a memory, as they are based on collections of derived inbred lines, with all genotypic and phenotypic information obtained in different experiments and by different actors referring to the same identical genotype; (ii) learn, from the analysis of the data integrated into the databases about the structure of genetic diversity and its relationship with phenotypic diversity and the environment; and (iii) improve and evolve, by correcting mistakes and making progressive adjustments of the sampling procedures according to the novel information that will be obtained.

Along with genotypic data, sub‐cores of nested core collections will be phenotyped in evaluation activities carried out by different actors, both internal and external to the INCREASE project consortium. The genotypic information and related genomic predictions will constitute the links between phenotypically characterized and non‐characterized accessions, as a very powerful tool to exploit the whole collection of genebank accessions, as far as the genotyping data are available. Genome‐wide association (GWA) analysis will be performed using conventional (Mackay et al., 2009; Ogura and Busch, 2015) and innovative machine learning (ML) approaches based on AI. Marker–trait associations will be identified to facilitate the exploration of germplasm collections not phenotypically characterized.

**Figure 2:**
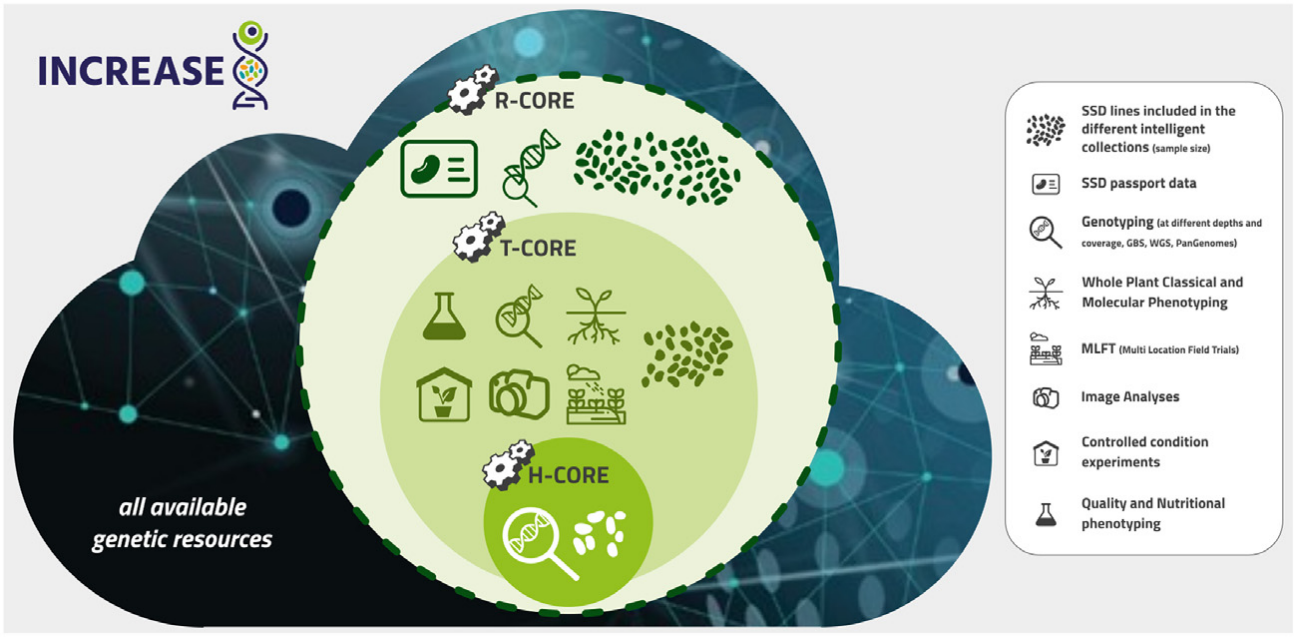
Schematic summary of INCREASE *Intelligent Collections* (ICs): Reference‐CORE (R‐CORE), Training‐CORE (T‐CORE) and Hyper‐CORE (H‐CORE) developed in INCREASE and activities that will be carried out on the different panels. Starting from all the available genetic resources for each of the four INCREASE legume species, R‐CORE will be constituted of thousands of single‐seed descent (SSD) lines, representative of the genetic resources of the species, with as complete and informative as possible passport data, and R‐CORE will undergo low‐coverage genotyping; T‐CORE will be constituted of a subset of R‐CORE (few hundreds of SSD lines) which will be involved in several phenotypic (including transcriptomic and metabolomic) and genomic characterizations (whole genome sequencing [WGS]); H‐CORE, the smallest sample (about 40–50 SSD lines, based on an evolutionary transect) will be in‐depth genotyped and phenotyped. All the genotypic data and information and related genomic predictions coming from the characterization of the ICs and of sub‐cores of nested core collections will be the link between phenotypically evaluated and non‐evaluated accessions from the *universe* of all available genetic resources (as far as genotyping data are available).

Thus, in detail, on each of the four INCREASE food legume crops, the project will develop nested core collections, including wild, landraces and cultivars (Figure 2):

(i) a Reference‐CORE (R‐CORE): the thousands (approximately 2000–4000) of genetically purified accessions obtained by SSD will be genotyped using a low‐coverage approach (e.g., genotyping by sequencing, exome capture); all of the R‐COREs of each species will be preserved as units in one or more genebanks that participate in the project, for long‐term and active conservation;
(ii) a Training‐CORE (T‐CORE): comprising a subset of R‐CORE, the Training‐CORE will be based on a few hundreds of genotypes (approximately 400–500) with comprehensive passport and georeferenced information; these will be used for phenotypic characterization (including transcriptomics and metabolomics) and genomic characterization, using whole‐genome sequencing;
(iii) a Hyper‐CORE (H‐CORE) of approximately 40–50 genotypes sampled on an evolutionary transect will be used for deep genomic and phenotypic characterization, to explore the ‘phenotypic space’ (i.e., reaction norm) and to identify key traits of interest for future phenotyping.

These ICs will be shared as a tool among genebanks, safety duplicated in the Svalbard Global Seed Vault (Fowler, 2008), and will co‐exist with the whole collections available in different genebanks to improve the overall management of the genetic diversity.

#### Criteria of selection of Intelligent Collections

As INCREASE aims to develop a worldwide sample of wild forms and landraces, as well as cultivars and improved varieties of different market classes and origins, with special attention given to germplasm adapted to European environments, including landraces cultivated on farm and crop wild relatives (CWRs), the selection of lines to be part of the ICs includes the following steps:

(i) removing as much as possible redundancy of accessions;
(ii) *selection of R‐CORE lines* (approximately 2000–4000 lines): (a) maximizing diversity for geographic origin and distribution, and environmental adaptation; (b) maximizing diversity on the basis of availability of previous genetic and phenotypic characterization; (c) including accessions with passport data (including geographical coordinates) available; (d) including accessions from historical and current collections of on‐farm and *ex situ* populations; (e) availability of SSD seeds; (f) availability of genomic data; (g) including approximately 20% of heterogeneous accessions to cover gaps and balance sampling structure and to include samples collected from heterogeneous populations (i.e., landraces recently collected);
(iii) *selection criteria for T‐CORE* (approximately 400–500 lines): (a) maximizing diversity present in R‐CORE; (b) availability of SSD lines seeds; (c) availability of genomic data. Part of the T‐CORE will include lines that will be used for field experiments; these lines will be exclusively chosen from the domesticated gene pool, and among those adapted to European agroecosystems; the remaining lines of the T‐CORE will be wild lines, domesticated lines not adapted to European environments and CWRs, which will be used mostly for controlled condition experiments. Thus, T‐CORE phenotyping will be based on different sub‐samples based on the type of experiment and the level of complexity of phenotyping (e.g., intercropping experiment using common bean and maize [*Zea mays*], drought‐controlled condition experiment for chickpea and common bean, etc.);
(iv) *selection of H‐CORE lines* (approximately 40–50 lines): this core collection will be used for deeper genotyping and phenotyping. Selection criteria will be to maximize the overall genetic diversity and to include highly characterized accessions (e.g., whole genome sequencing [WGS]). Also, the H‐CORE’s characterization will be based on the entire set of lines or sub‐samples depending on the type of experiments.

### INCREASE IC characterization

#### Classical and molecular phenotyping

Having assembled the above‐described ICs they will be subjected to extensive phenotyping at three levels: (i) multi‐location field trials (MLFTs), (ii) metabolomics and transcriptomics and (iii) root traits, disease and symbiosis as detailed here below.

Comprehensive agronomic and morphological phenotyping of the T‐CORE accessions will be performed under field conditions using standard phenotyping to assess the adaptation and the agronomic performances of the genotypes of the four species along with the effect of genotype versus environment (G × E) interaction (Beleggia et al., 2013). Field trials will be conducted for each species in three locations for 2 years with three replications along with the use of repeated checks (10%) to take into account the field heterogeneity. Moreover, for common bean and lentil, an additional field trial will be conducted in Canada thanks to the collaboration with the INCREASE third party, the University of Saskatchewan. An intercropping experiment on common bean (with maize) will additionally be performed using a subset of T‐CORE accessions and subjected to agronomic and morphological phenotyping (in two locations for 2 years).

It is envisaged that during MLFTs several phenotypic traits will be scored (e.g., growth habit, plant height, flower color, along with important traits related to grain production, days to flower and maturity, seed and pod number and weight, etc.), applying crop‐specific phenotyping protocols based on standard crop descriptors, also included in crop ontologies (Cortinovis et al., 2021; Guerra‐García et al., 2021; Kroc et al., 2021). In addition, we will use smart phenotyping tools, based on image analysis for phenotypic data on traits of interest (e.g., seeds, flower, pods and leaves). Image analysis based on deep learning will be used to analyze the images, allowing a deep evaluation of seed color, seed patterns, seed size and seed shape, in a precise and rapid manner. Furthermore, root traits will be recorded in the field (‘*shovelomics*’; Trachsel et al., 2011) using a subset of the lines present in the MLFTs (which will include the domesticated genotypes of the H‐CORE).

A large‐scale and comprehensive metabolic profiling across the four legume species and many genotypes across different environmental conditions on both leaf and seed tissues will be conducted. Considering the complexity of legume species metabolism, we will use different approaches to ensure metabolite annotation: (i) using an in‐house library (with >3500 compounds) and (ii) using the accurate *m*/*z* peaks in full scan and MS2 produced by Orbitrap high‐resolution mass spectrometry using a Q Exactive^TM^ Focus Hybrid Q‐Orbitrap (Thermo Scientific, Waltham, MA, USA) to match online databases (e.g., m/zCloud, Chemspider, GNPS) (Aksenov et al., 2021; Aron et al., 2020) for peak annotation. In addition, the MS and MS2 spectra will be compared with previously identified compounds and published articles in plants and specifically the targeted species. The metabolite output data will be reported and documented in Excel files following our recent recommendation standard (Alseekh et al., 2021a) and will be available in the project respiratory database. In parallel to the metabolite profiling, we will perform transcriptomic analysis on leaf tissue of the same materials. The transcriptomic and metabolic analysis will provide essential information to be integrated with genomic data in order to understand the molecular mechanisms associated to phenotypic responses and G × E of plant genetic resources and to highlight quantitative loci underlying metabolic and transcriptomic variation via the performance of network analyses (Beleggia et al., 2016; Bellucci et al., 2014; Perez de Souza et al., 2019). We intend to identify transcripts strongly correlated with the abundance of given metabolites across tissues and genotypes and further correlate metabolite content with physiological data, to define the positively and negatively correlated phenotypic traits with signal metabolites (Guillamon et al., 2008; Zhu et al., 2018). In addition, we will use the metabolite data collected from different environments as biomarkers to predict plant growth and behavior. Finally, transcriptome/metabolome data will be used to guide gene functional annotation and elucidation of species‐ and/or tissue‐specific metabolic pathway structures.

Crop plants produce compounds that affect their market quality, taste and nutritional value, as well as numerous compounds that contribute to human health. Therefore, in addition to metabolomics and transcriptomic analysis and taking advantages of the metabolomics profiling, INCREASE will determine several quality traits including antioxidant content as well as that of other health‐related compounds and anti‐nutritional metabolites, such as seed alkaloids (Muzquiz et al., 2012; Wink et al., 1995). Within the objectives of INCREASE, our aim is to elucidate the underlying cellular, biochemical and molecular mechanisms associated with nutritional quality aspects under different environments; the candidate genes will be identified in addition to rational strategies for the development and selection of genotypes with high nutritional properties.

The disclosure of less‐adapted material, such as CWRs, in addition to the genomic characterization, will primarily focus on analysis of disease resistance, nutritional and quality traits, along with root traits and tolerance to abiotic stresses, key elements in the breeding process nowadays, for which CWRs are undoubtedly a source. Special emphasis will be given to analysis of molecular diversity within gene families known to play important roles in plant–microbe interactions (Meziadi et al., 2016; Roy et al., 2020). Metabolomic analysis will be a highly valuable tool for such characterization.

Phenotyping under controlled conditions will also be performed using automated high‐throughput systems.

A specific test will be conducted under controlled conditions for root traits, using paper blotting for chickpea, common bean, lentil and lupin on the T‐CORE including those evaluated for root traits in MLFTs. The same set of lines will be evaluated under control conditions for disease resistance to anthracnose, white mold, powdery mildew and ascochyta in common bean and lentil to combine association genetics and resistance (*R*) gene enrichment sequencing.

The ability to establish symbiosis with rhizobia, as well as the efficiency of biological nitrogen fixation and competitiveness for nodulation, will be assessed in plant inoculation assays in controlled environmental conditions, whereas validation studies will be performed in chickpea and common bean in order to screen germplasm tool to maximize diversity in symbiotic responses.

A proper understanding of the mechanisms that govern drought tolerance in plants is therefore of the utmost importance in the context of climate change mitigation. INCREASE will study drought tolerance in chickpea and common bean, employing non‐invasive precision phenotyping under controlled conditions (Chen et al., 2014; Dhanagond et al., 2019). In these experiments several phenotypic and morphological traits will be scored, including days to flower and maturity, growth habit, plant height, seed number, seed weight and flower color, and leaf samples will be collected for metabolomic and transcriptomic analysis as described for field trials. In addition, we will evaluate the effects of water stress on plant and leaf photosynthesis.

#### Genomics and genotyping

The most recent and advanced sequencing technologies will be applied to sub‐samples of H‐CORE genotypes to develop pan‐genomes of common bean and lentil and obtain new high‐quality *de novo* whole genome sequences (i.e., a contiguous, haplotype‐resolved representation of the entire genome) for the four INCREASE species. The already available genotypic data of all the species (generated by the INCREASE consortium and already present in the public domain) will be exploited and integrated, including the reference genomes of all the species.

High‐quality whole genome assemblies and pan‐genomes (core and dispensable genes) will be developed and we will use several approaches to exploit the structure of the genetic diversity and identify the functional diversity that could have a major role in the conservation, adaptation and improvement of food legumes. Nucleotide and structural variations (inversions, deletions, insertions) will be identified in order to highlight and explore crucial functional roles and associations with several phenotypic traits (Alseekh et al., 2020; Fernie and Aharoni, 2019; Jayakodi et al., 2020; Liu et al., 2020; Varshney et al., 2019; Yang et al., 2019). Moreover, we will perform advanced comparative genomics across the Fabaceae family in order to gain fundamental insights into the structure of functional genetic diversity and its evolution across a broad evolutionary scale.

The SSD lines for all the ICs will be subjected to different levels of genome sequencing, depending on core collection and genome size: R‐CORE genotyping will allow a genetic characterization of a large amount of plant genetic resources for which we could predict the phenotypic performances using the link between genotype and phenotype in relation to the environmental variation that will be established using phenotypic data from the T‐CORE and H‐CORE.

Based on the large amounts of data produced we will investigate the demography and selection shaping neutral genetic diversity and functional variation of crops; we intend also to identify the genetic structure that if unknown would result in failure in identification of selection signals and in the identification of spurious genotype–phenotype associations.

We will undertake genome‐wide scans to identify candidate polymorphisms (outliers) involved in local adaptation and rely on demographic scenarios inferred and ML algorithms to detect footprints of selection. In addition, we also intend to associate allele frequencies to environmental variation by taking latitude and longitude as proxies. During these field evaluations of the association panel, traits such as grain production will be taken as proxies for fitness.

In order to seek adaptive variation and its determinants, we will further identify the alleles underlying the ability of genotypes to perform in any given environment, thanks to GWA studies applied to both the core and pan‐genomes.

Based on the T‐CORE, we aim to establish prediction equations for different traits × environment combinations, using most recent methods developed to take genetic structuring into account. We will additionally develop implementation routines for material sequenced at low depth and predict phenotypes for each R‐CORE line. We will further test how the inclusion of GWA results improves prediction models. Moreover, in common bean and lentil we will study Nucleotide‐binding Leucine‐rich Repeat (NLR) *R* gene clusters using resistance‐gene enrichment (Jupe et al., 2013) and by applying long‐read sequencing to allow us to uncover complex NLR clusters and characterize the pan‐NLRome (Van de Weyer et al., 2019).

Single‐seed descent‐purified genotypes derived from on‐farm conservation, using at least two genotypes per population (farmer’s field), will be used to estimate the diversity within the population; population sequencing using Pool‐Seq (pool sequencing, i.e., sequencing by pooling DNA samples) will be carried out to determine the allelic diversity within populations and within heterogeneous accessions, to follow, in the future, their changes over time and to test strategies to infer the haplotypic structure of heterogeneous accessions from Pool‐Seq.

Accessions are composed of a mixture of genotypes and current breeding schemes use one or a few SSDs out of an accession to represent it, capturing only a small fraction of the existing adaptive diversity within this accession. On the other hand, genetic diversity within one accession is usually described by allele frequencies, but characterization of the mosaic of haplotype blocks contained in an accession would increase the predictive power of their breeding potential. An example of such an application would be to screen uncharacterized accessions for haplotypes known to be associated with traits of interest. Another would be the improved monitoring of haplotype frequencies in *ex situ* collections. Our goal will be to access such haplotype information using both sequencing of pools and multiple individuals (Pool‐Seq) from a single accession, alongside the collection of haplotypes recovered from the H‐CORE.

Genomics data will be used to: (i) reveal accessions with mismatching passport information and potentially correct those, and to reduce duplicated accessions and redundancy as in the barley (*Hordeum vulgare*) IPK collection (Milner et al., 2019); (ii) define core collections capturing a high diversity of the total collection to be in‐depth phenotypically and genotypically characterized (Milner et al., 2019; Wambugu et al., 2018); (iii) identify unique germplasm that needs particular attention in conservation (Gowda et al., 2013); and (iv) strictly monitor the multiplication of accessions (Mascher et al., 2019). Finally, advanced molecular methods will be applied to assess the levels and values of heterogeneity in germplasm accessions and in populations conserved on‐farm (Frankin et al., 2020; Gouda et al., 2020). Genomics data will be used to sample highly variable sets of accessions for target genes associated with traits of interest.

All results from the data analysis will be imported into the databases, with complementary visualization tools provided via user‐friendly web applications (Sanderson et al., 2013, 2019).

## INCREASE INNOVATION AND NEW ROOT IN GERMPLASM MANAGEMENT

INCREASE will ultimately analyze data amassed in the project to deliver new knowledge that will be available for users thought the web portal implementing visualization tools and allow the identification of the most useful germplasm. Based on the deep IC characterization, we aim to reveal the structure and distribution of the preserved legume plant genetic resources, to solve redundancies, duplications and gap issues, to correct wrong passport data and, particularly, to predict the genotypic values of accessions that will be genotyped only at low coverage. This will allow users to sample a set of accessions that might maximize their expectations, considering their specific objectives (e.g., identify accessions with high frequency of genotypes adapted to a given environment, with high protein content or low abundance of an anti‐nutritional factor). INCREASE will verify the potential of direct genotyping of heterogeneous accessions from pooled DNA samples, as a way of by‐pass SSD purification using R‐CORE haplotype data as the reference dataset, inside an open and traceable data‐sharing policy.

INCREASE will additionally tailor phenotyping approaches based on the needs of the users (i.e., breeders, farmers, agri‐food and non‐food industries, gardeners, consumers), not only considering conventional traits, but also accounting for specific features of the value chain (e.g., food quality, nutrition value) and of the target agro‐ecosystem (e.g., crop rotation, intercropping, biological treatments of crops, sustainable agriculture under the aggravating conditions of climate change), with the possibility to explore *genotype* *×* *environment* interactions for adaptation to different environmental conditions. It is intended that this will not only encompass agronomic performance, but also include information concerning nutritional content and quality traits. Moreover, the combined use of transcriptomic and metabolomic approaches and their associated networks will allow identification of putative genes responsible for phenotypic plasticity (Alseekh et al., 2017, 2021b; Beleggia et al., 2016; Liu et al., 2021; Zhu et al., 2018). Artificial intelligence, and in particular deep learning techniques based also on multispectral images, will support the overall information discovery process by collecting annotated datasets and modeling major phenotypic effects.

INCREASE will obtain high‐quality genotypic data anchored on pan‐genome assemblies, with the possibility to exploit the structural variations of the molecular diversity of these food legumes. This could be particularly important, as many structural variations have major phenotypic effects, such as disease *R* gene clusters, which can be difficult to characterize as they can have a highly repeated structure (Chen at al., 2018; Gao et al., 2016; Richard et al., 2018).

In INCREASE a new data structure and web portal for data analysis will be designed within the modern Big Data Analytics concept and with a data sharing layer able to overcome the described limitation by providing different tools, for different users, but embedded within a powerful data architecture of huge computational power.

The web portal will provide a framework for the integrated tools, allowing users to define their own subsets (collections) based on the data stored in the project’s databases. It will provide features to navigate between and combine the different search results of the domains (phenomics, genomics, metabolomics, taxonomy and georeferences). The concept of the IC should make it possible to suggest suitable candidates of plant genetic resources to the user that match his or her requirement profile. However, experience from existing platforms, such as BRIDGE for barley (König et al., 2020) or the ‘KnowPulse’ (Sanderson et al., 2019) and the BeanAdapt portal, will be incorporated into the crop‐specific solutions.

In order to make the complexity of different crops, data domains and, last but not least, different user groups manageable, the users will be given guidance by a couple of options. They will be supported by online tutorials, hands‐on videos that explain the respective tools. Basic functions, such as navigation, will be explained intuitively through an interactive tutorial. As the concept of user‐specific collections will play a central role, the provision of pre‐defined collections will simplify the exploration of the different tools. As part of the stakeholder support, webinars addressing specific topics are also a conceivable option.

By focusing on data management and sharing, the combination and use of data generated by users during their activities will be facilitated. On the one hand, the incorporation of the user’s data will make it possible to carry out an analysis tailored to the user, and on the other hand, it will create a dynamic management of plant genetic resource data.

In this way, INCREASE will blaze the trail for integrated and dynamic conservation management of legume genetic resources across European and non‐European institutions and initiatives. INCREASE will also support seed distribution management by evolving the Easy‐Standard Material Transfer Agreement (eSMTA) concept to a smart contract (Brink and Van Hintum, 2020), thus rendering genetic material sharing facile in a validated digital process.

## CONCLUDING REMARKS

INCREASE plans to establish an open space for the efficient and effective conservation and use of food‐legume genetic resources and will generate, analyze and integrate massive, novel and already available genotypic and phenotypic information by the development of improved databases and innovative visualization tools. Indeed, we will improve genebank standards for data management and develop a central data management infrastructure and an expert portal, which will connect plant genetic resources with precise and high‐quality genotypic and phenotypic information along with widely homogenized passport data. This will facilitate plant genetic resource exploration and we are convinced that including research scientists, breeders, genebank curators, farmers, agri‐food and non‐food industries and consumers will dramatically boost the competitiveness of legumes in the European agriculture and food sector, having a major impact on economy and society as well as addressing the need for enhancing the production of plant protein crops in Europe.

## PARTICIPATORY APPROACH AND OPEN SCIENCE

INCREASE will enhance management and use of plant genetic resources by implementing a participatory approach to acquire relevant plant genetic resources and related information worldwide, to expand the quality and quantity of seed and information resources, along with the interests of European users. The project involves a wide range of stakeholders (R&D, value chain, scientific research, civil society, environmental non‐governmental organizations (NGOs), schools, citizens) interacting with each other under the coordination of INCREASE. The aims are to create and work within a ‘legume research community’, genebanks, plant breeders, plant and crop scientists, farmers (in conventional and organic agriculture), gardeners, seed suppliers, food industry, database and computational experts, NGOs related to environmental conservation and associations of citizens that will contribute to the project and, at the same time, benefit from the data produced and from the outcomes reached, enhancing the management and use of genetic resources on these important food legumes and maximizing the project impact.

**Figure 3:**
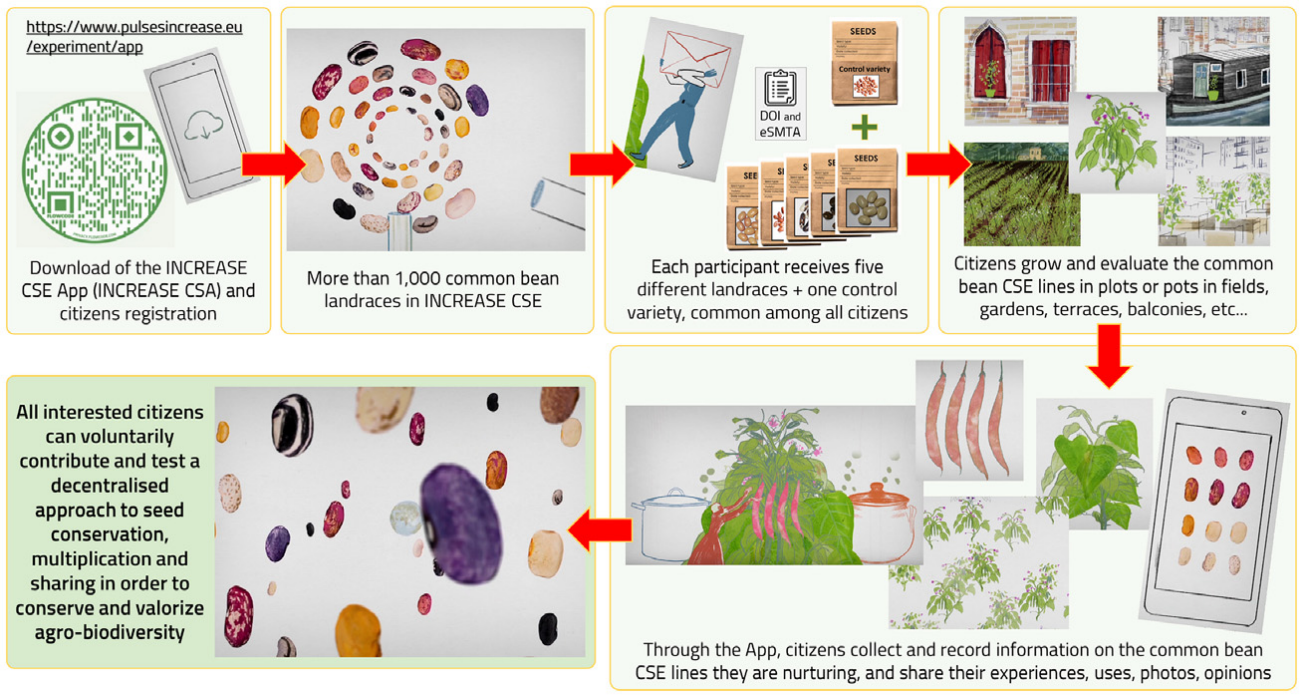
INCREASE citizen science experiment (CSE, illustration by Daniele Catalli). Registered CSE participants, using the expressly developed and constantly updated INCREASE CSE app, will be involved in the conservation, evaluation and valorization of the common bean genetic resources and will test the INCREASE decentralized approach to genetic resource conservation, sharing and valorization.

Moreover, within INCREASE, a citizen science experiment on common bean has recently been launched (Irwin, 2018; van Etten et al., 2019; Würschum et al., 2019; www.pulsesincrease.eu/experiment; Figure 3): external participants, throughout the registration and the use of a dedicated app (Increase CSA, Ubisive srl, from the Google Play Store or the App Store), can exploit a subset of genotypes from R‐CORE (about 1000 genetically purified accessions that have been genotyped within the project BEAN_ADAPT; ERA‐CAPS 2° call, 2014) to contribute to conservation, evaluation and distribution of food‐legume plant genetic resources using seed image recognition as the validating tool; we included eSMTA generation and reporting functions and digital agreements, in a collaboration with the FAO ITPGRFA, INCREASE partner, a solution, designed for the general public, that will facilitate sharing seeds in a legal framework and could be also adopted by expert users that often find the actual SMTA procedures uneasy and time consuming, enhancing the actual state of the art of the SMTA by introducing a digitalized process based on smart contracts easily managed by end‐users on a mobile application.

## INCREASE CONSORTIUM DESCRIPTION

**Figure 4:**
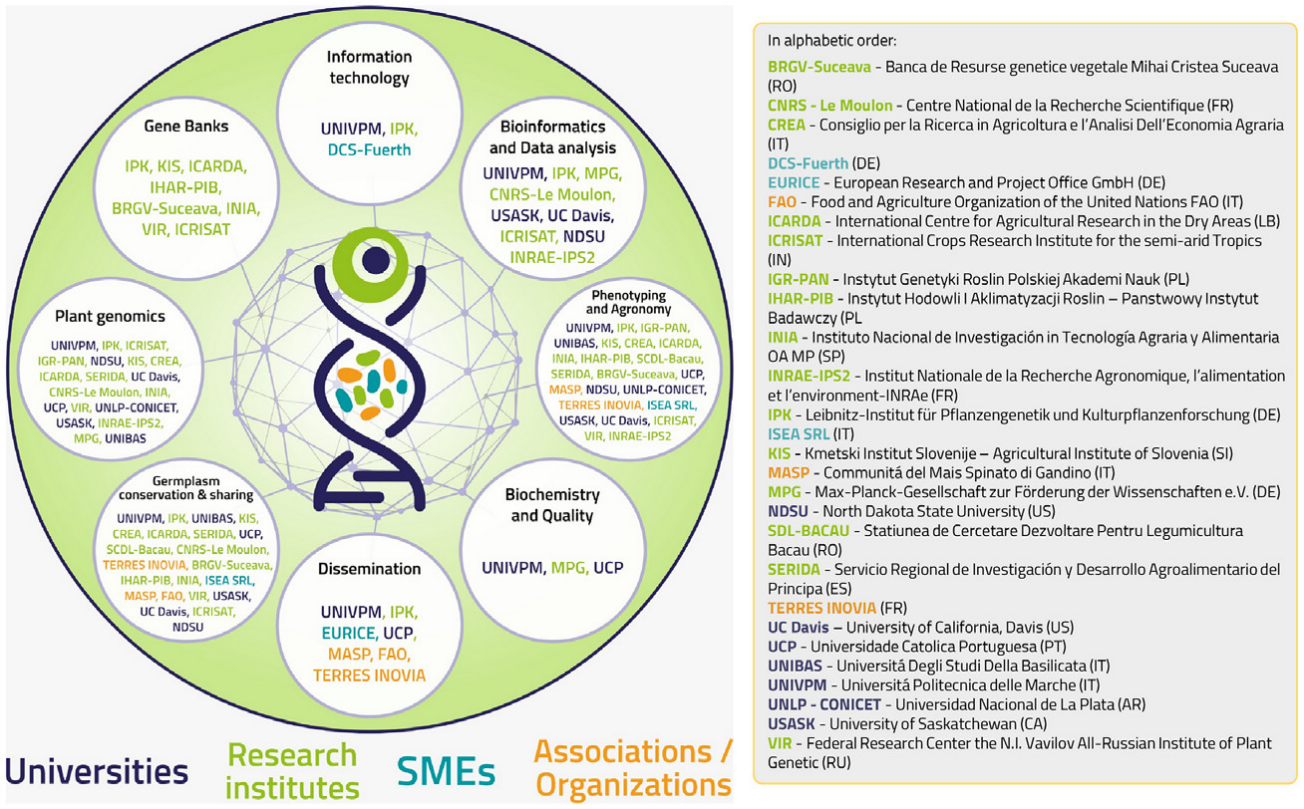
Interdisciplinary expertises and roles of the INCREASE partners. *UNIVPM*: coordination, common bean crop leader, involved in all activities, responsible for IC development and blockchain approach for decentralized conservation. In alphabetic order: *BRGV‐Suceava*: best practices definition, assembly of collections, data curation and germplasm management; *CNRS‐LeMoulon*: coordination of data analyses, from pan‐genome development to genetic diversity, allele discoveries, GxE interactions and genomic predictions; *CREA*: data production and *in silico* analysis of genomic regions involved in nitrogen fixation; *DCS‐Fuerth*: SME, blockchain infrastructure design, innovation and dissemination; *EURICE*: SME, all project management aspects, innovation, communication and dissemination; *FAO*: conservation and exchange of genetic material, ethics advisory and technical requirements, dissemination and innovation; *ICARDA*: best practices definition, assembly of collections, phenotyping and seed increase, data curation and germplasm management, focusing on lentil and chickpea; *ICRISAT*: genomic data production and analyses, development of central data management infrastructure; *IGR‐PAN*: lupins crop leader, genetic resources management and multi‐omics’ characterization; *IHAR‐PIB*: best practices definition, assembly of collections and phenotyping and seed increase; *INIA*: chickpea crop leader, best practices definition, assembly of collections, phenotyping, seed increase, data curation and germplasm management; *INRAE‐IPS2*: focus on common bean, data production and analyses for identification of disease resistance genes using Ren‐Seq; *IPK*: central data infrastructure and collection, curation and dissemination of the data, new guidelines for germplasm management; *ISEA SRL*: SME, field trial and phenotyping; *KIS*: best practices definition, phenotyping, seed increase, data curation and germplasm management; *MASP*: assembly of collections, innovation and dissemination; *MPG*: coordination of data production, sequence analysis and phenotyping, generation of metabolomic and transcriptomic data; *NDSU*: focus on common bean, germplasm and genotypic information, data analyses, SNP discovery; *SDL‐BACAU*: assembly of collections, phenotyping and seed increase; *SERIDA*: assembly of collections, seed increase and field trials; *TERRES INOVIA*: coordination of stakeholders’ interface, dissemination and innovation; *UC Davis*: focus on chickpea, nitrogen fixation, abiotic and biotic stresses, genetics and genomics of wild trait introgression; *UCP*: data production, molecular phenotyping, nutritional and technological quality assessment; *UNIBAS*: lentil crop leader, coordination of phenotypic data production, cores seed increase, SSD development and trials; *UNLP‐CONICET*: analyses of genes involved in symbiotic interaction with rhizobia; *USASK*: focus on chickpea, germplasm and genotypic information, data analyses; *VIR*: DNA and herbarium samples, development of germplasm management guidelines.

The INCREASE consortium is composed of 25 partners, highly diverse in the type of organization and highly complementary in expertise (Figure 4). The INCREASE consortium comprises:

(i) the international organization FAO, which has a major role in promoting the sustainable use and sharing of plant genetic resources and in the implementation of the related policy;
(ii) two associations of stakeholders (TERRES INOVIA and MASP);
(iii) three subject matter experts (SMEs) from the seed (ISEA SRL), IT (DCS‐Fuerth) and service (EURICE) sectors;
(iv) 19 research institutions, spread across 12 countries (namely Italy, Germany, Poland, Slovenia, Lebanon, Spain, France, Romania, Portugal, Russia, Argentina and India), including European and international universities, institutions focusing on fundamental research and research institutions focusing on agricultural research, including five EU and three non‐EU genebanks.

INCREASE brings together and coordinates the main international efforts towards the acquisition of genomic information and phenotypic evaluation of hundreds of accessions of the four target species. The involvement of non‐EU partners expands the scope and ambition of INCREASE by integrating a significative amount of additional data and plant genetic resources (e.g., *de novo* genome sequences, genomic and phenotypic data) with SSD‐purified accessions.

## AUTHOR CONTRIBUTIONS

All authors contributed to the writing of this manuscript.

## CONFLICT OF INTEREST

All authors of this manuscript declare no conflict of interest.
